# The human blood DNA methylome identifies crucial role of β-catenin in the pathogenesis of Kawasaki disease

**DOI:** 10.18632/oncotarget.25305

**Published:** 2018-06-19

**Authors:** Kuang-Den Chen, Ying-Hsien Huang, Mindy Ming-Huey Guo, Tzu-Yang Lin, Wei-Teng Weng, Hsiang-Jen Yang, Kuender D. Yang, Ho-Chang Kuo

**Affiliations:** ^1^ Department of Pediatrics and Kawasaki Disease Center, Kaohsiung Chang Gung Memorial Hospital and Chang Gung University College of Medicine, Kaohsiung, Taiwan; ^2^ Institute for Translational Research in Biomedicine, Liver Transplantation Center and Department of Surgery, Kaohsiung Chang Gung Memorial Hospital and Chang Gung University College of Medicine, Kaohsiung, Taiwan; ^3^ Department of Pediatrics, Mackay Memorial Hospital, Taipei, Taiwan; ^4^ Institute of Biomedical Sciences, Mackay Medical College, New Taipei City, Taiwan; ^5^ Institute of Clinical Medicine, National Yang-Ming University, Taipei, Taiwan

**Keywords:** β-catenin, CD40, CD40 ligand, methylation, Kawasaki disease

## Abstract

Kawasaki disease (KD) is a type of acute febrile vasculitis syndrome and is the most frequent cause of cardiac illness in children under the age of five years old. Although the etiology of KD remains largely unknown, some recent genome-wide studies have indicated that epigenetic factors may be important in its pathogenesis.

We enrolled 24 KD patients and 24 non-KD controls in this study to access their DNA methylation status using HumanMethylation450 BeadChips. Another 34 KD patients and 62 control subjects were enrolled for expression validation. Of the 3193 CpG methylation regions with a methylation difference ≥ 20% between KD and controls, 3096 CpG loci revealed hypomehtylation, with only 3% being hypermethylated. Pathway buildup identified 11 networked genes among the hypermethylated regions, including four transcription factors: nuclear factor of activated T-cells 1, v-ets avian erythroblastosis virus E26 oncogene homolog 1, runt related transcription factor 3, and retinoic acid receptor gamma, as well as the activator β-catenin. Ten of these network-selected genes demonstrated a significant decrease in mRNA in KD patients, whereas only CTNNB1 significantly decreased in correlation with coronary artery lesions in KD patients. Furthermore, CTNNB1-silenced THP-1 monocytic cells drastically increased the expression of CD40 and significantly increased the expression of both CD40 and CD40L in cocultured human coronary artery endothelial cells.

This study is the first to identify network-based susceptible genes of hypermethylated CpG loci, their expression levels, and the functional impact of β-catenin, which may be involved in both the cause and the development of KD.

## INTRODUCTION

A form of acute febrile systemic vasculitis, Kawasaki disease (KD) was first described by Kawasaki et al. in 1974 [1]; however, its origins are still generally unknown today. This disease affects children around the world, particularly those under the age of five years old. In general, KD patients clinically present with a prolonged fever for more than 5 days and have at least four of the following five major symptoms: diffuse mucosal inflammation, bilateral non-purulent conjunctivitis, cervical lymphadenopathy, indurative angioedema of the hands and feet, and polymorphous skin rashes, as was previously described [2]. The most serious complication arising from KD is coronary artery lesions (CAL), including myocardial infarction, coronary artery dilatation, coronary artery fistula, and coronary artery aneurysm [3]. In fact, KD is currently the leading cause of acquired heart disease in children in developed countries [4], and approximately 20-25% of untreated KD children develop coronary artery aneurysms.

While the exact origins of KD remain uncertain, growing evidence has suggested that the development of KD may be similar to an immune/autoimmune process [5]. Some researchers have suggested that KD may be prompted by an infectious agent in individuals with a genetic predisposition but not actually an infectious disease. Previous genome-wide association studies (GWAS) have shown that many genes are associated with susceptibility to KD, including FCGR2A [6], CD40 [7], BLK [8], and ITPKC [9]. The GWAS of various geographic areas had different results, with only CD40 and BLK being consistent in reports from both Japan and Taiwan. Environmental factors, epigenetic variants (FCGR2A), genetic factors, infectious agents, and lifestyle (such as the intake of food containing mercury), etc. have all been reported to be associated with KD, thus suggesting that KD is a complex disease. Previous studies have found that epigenetic regulatory mechanisms are correlated with various diseases. Using data from our previous studies, we performed DNA methylation array using the Infinium HumanMethylation27 BeadChip array to evaluate the association between genomic hypomethylation of FCGR2A and the susceptibility to KD and intravenous immunoglobulin resistance [10]. The purpose of this study is to examine more detailed epigenetic changes in the susceptibility and pathogenesis of KD using HumanMethylation450 BeadChip, as well as to investigate potential mechanisms using a cell-cell interacting vasculitis model.

## RESULTS

### DNA methylation dynamics in KD patients’ peripheral blood

**Figure 1:**
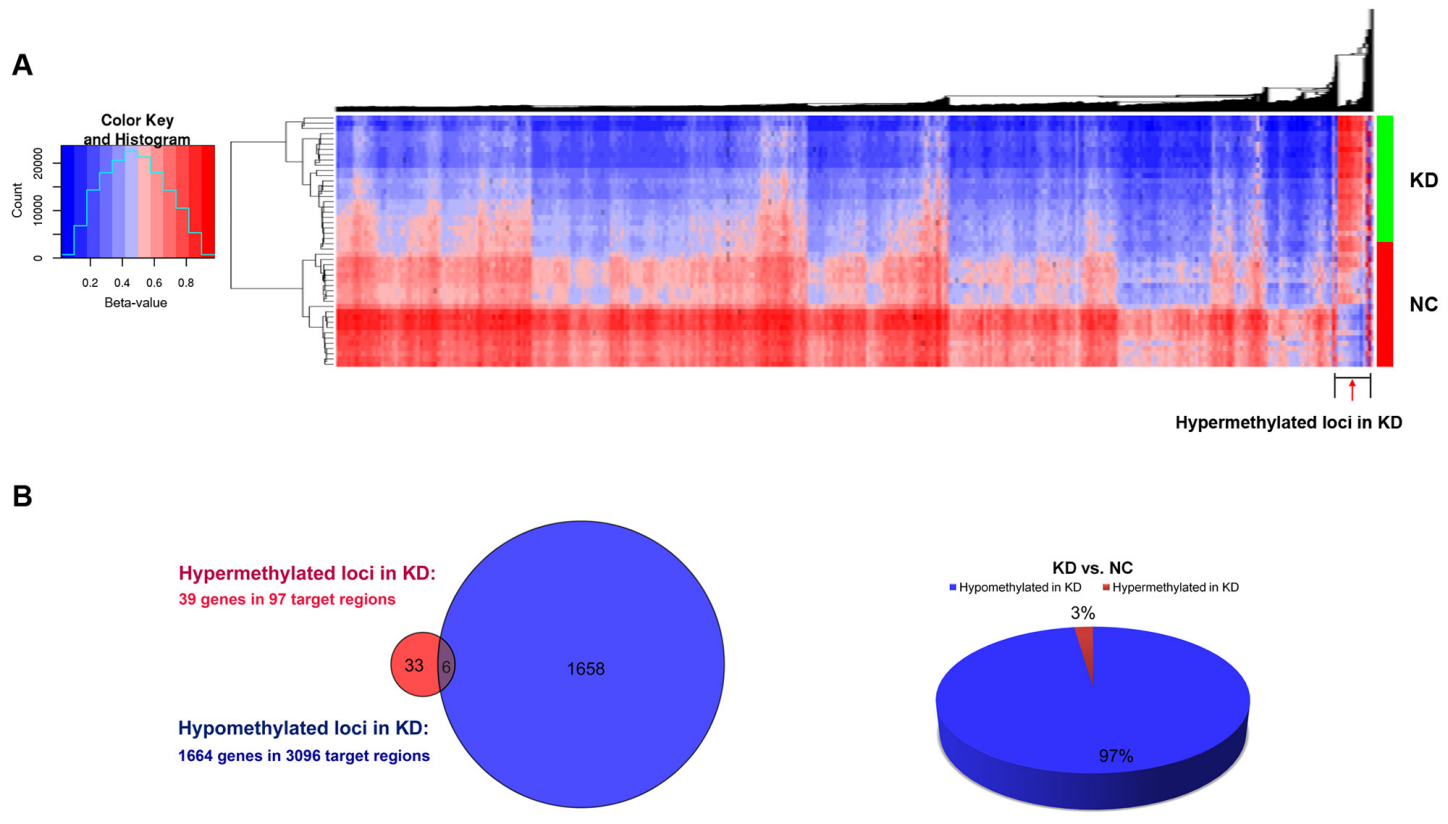
Differential methylation of individual CpG loci in peripheral blood **(A)** Unsupervised two-way hierarchical clustering and heat map of genomic regions that include CpG di-nucleotides with a methylation difference of ≥20% found between 24 Kawasaki disease patients (KD) and 24 normal control subjects (NC). **(B)** The Venn diagram on the left shows the number of associated genes of hypermethylated CpG loci (red) and hypomethylated CpG loci (blue) in KD patients, with overlapping genes in purple; the pie chart on the right depicts the percentage of hypermethylated CpG loci (red) and hypomethylated CpG loci (blue) in KD patients.

We took peripheral blood samples from a total of 48 individuals (24 KD patients and 24 normal controls; see Table 1 upper panel) and subjected them to access DNA CpG methylation state using Infinium HumanMethylation450 BeadChips. This array consists of probes that determine the site-specific methylation status of more than 450K CpG loci across the human genome. In total, 1,703 genes were found distributed among 3,193 CpG regions (DMRs) with a methylation difference ≥ 20% between KD patients and normal control subjects (NC). Most DMRs were hypomethylated (3,096 target CpG loci) in the KD group, which corresponded to 1,664 imprinted genes, whereas only 3% DMRs (97 CpG loci) were hypermethylated across 39 genes (Figure 1).

**Table 1:** Basal characteristics of patients with Kawasaki disease (KD) and control subjects

Characteristics	KD Patients	Controls	*p*-value
**Primary Set for M450 Array**			
Number of subjects	24	24	-
Male/Female	13/11	12/12	0.778
Age at study (years)	1.91±1.52	2.24±1.07	0.401
Age range	0 – 7	0 – 6	-
**Validation Set**			
Number of subjects	34^ǂ^	62	-
Male/Female	16/18	32/30	0.882
Age at study (years)	1.87±1.58	2.46±1.95	0.088
Age range	0 – 7	0 – 10	-

^ǂ^The number included 13 CAL and 21 non-CAL KD patients.

### Clusters of methylation signatures in KD with network-based gene enrichment analysis

**Figure 2:**
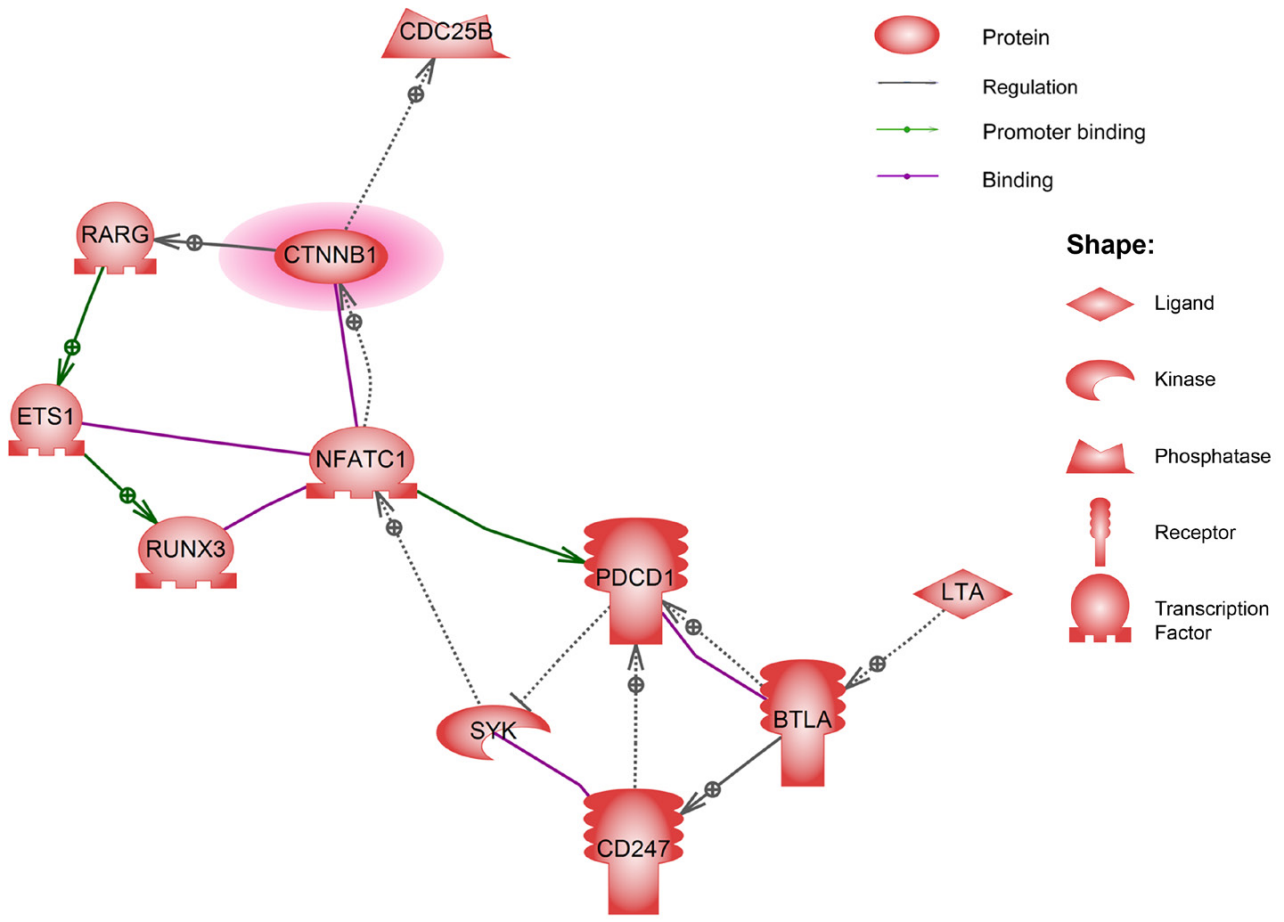
The direct interaction network of the genes identified from imprinting the hypermethylated regions in KD patients

To improve the understanding of the biological context of the changes in the corresponding genes of DMRs in KD, 39 genes imposed on 97 hypermethylated loci and 1,664 genes imposed on 2,096 hypomethylated loci were additionally analyzed using Ariadne’s pathway buildup for direct interaction and were further enriched using sub-network analysis (SNEA) to identify the respective putative networks. The enriched sub-networks, which included a set of single seed genes with target genes that correlated with the seed among these imprinted genes, were presented in a network layout, as shown in Figure 2, in which an enriched network containing 11 genes imposed on the rare hypermethylated regions in KD patients was connected. In a complex formed by the four transcription factors of nuclear factor of activated T-cells 1 (NFATC1), v-ets avian erythroblastosis virus E26 oncogene homolog 1 (ETS1), runt related transcription factor 3 (RUNX3), and retinoic acid receptor gamma (RARG), we considered the transcription activator β-catenin (CTNNB1) a core regulator in the sub-network, which normalizes both cell cycle progression and the severity of tissue inflammation. Table 2 (upper panel) shows the corresponding CpG regions identified using SNEA. However, we observed no significant gene-gene correlation with direct interaction among the abundant 1,664 genes imposed on the major hypomethylated regions in KD patients.

**Table 2:** Hypermethylated CpG loci selected using network-based enrichment analysis and methylation levels (%) that were technically validated by pyrosequencing in KD patients (n = 24) compared to normal control subjects (NC; n = 24)

Hypermethylated CpG loci of network-enriched genes						
CpG loci ID	Gene Symbol	Genomic Location	Strand	Functional Location	Delta-Beta	*p*-value
cg24157392	BTLA	3:113700663	F	Body	0.25195	1.60E-08
cg09032544	CD247	1:165753919	R	Body	0.31832	7.70E-04
cg07786657		1:165754257	R	Body	0.36159	5.60E-05
cg14165142	CDC25B	20:3726655	R	Body	0.25253	1.10E-08
cg02737268		20:3728182	F	Body	0.42308	1.90E-09
cg15421087	CTNNB1	3:41215859	F	TSS200	0.25553	3.20E-05
cg03295554	ETS1	11:127900660	F	Body	0.26371	1.00E-05
cg26404422		11:127872220	F	Body	0.26570	3.40E-04
cg26348243	LTA	6:31648440	R	5′UTR	0.28315	7.60E-04
cg14441276		6:31647714	R	TSS1500;TSS200	0.25889	2.40E-06
cg02402436		6:31648030	F	TSS200;1stExon;5′UTR	0.26393	1.70E-09
cg21999229		6:31647993	F	TSS200;1stExon;5′UTR	0.26700	1.50E-05
cg16219283		6:31647981	F	TSS200;1stExon;5′UTR	0.27092	9.00E-12
cg14597739		6:31647977	F	TSS200;1stExon;5′UTR	0.28225	2.50E-08
cg17169196		6:31648005	F	TSS200;1stExon;5′UTR	0.28398	6.90E-06
cg09621572		6:31647952	F	TSS200;1stExon;5′UTR	0.33790	3.70E-10
cg14437551		6:31647965	F	TSS200;1stExon;5′UTR	0.36865	5.10E-06
cg22324981	NFATC1	18:75384481	F	Body	0.30353	4.00E-10
cg16308790		18:75326961	R	Body	0.28664	4.50E-06
cg03889044	PDCD1	2:242450772	F	TSS1500	0.26858	3.10E-10
cg17322655		2:242450800	F	TSS1500	0.27499	1.90E-08
cg20805133		2:242450865	R	TSS1500	0.36842	6.60E-05
cg09993145	RUNX3	1:25164492	R	TSS1500	0.38990	4.20E-04
cg13461622		1:25163972	R	1stExon;5′UTR	0.27998	2.20E-09
cg03961551		1:25124317	R	Body	0.29300	4.30E-06
cg14054883	SYK	9:92659288	R	Body	0.26680	1.60E-08
cg20059012	RARG	12:51899421	F	Body	0.27935	7.70E-07

Validation results of the methylation levels (%)								
Gene Symbol	CpG loci	Methylation (%)						p-value
		NC (N=24)			KD (N=24)			
		Mean	SD	95% CI	Mean	SD	95% CI	
NFATC1	1	29.75	9.11	24.60–34.90	49.79	8.34	46.45–53.13	1,37E-6^***^
	2	27.27	8.22	22.51–31.82	44.42	8.73	40.92–47.91	3.01E-6^***^
	3	38.25	11.82	31.57–44.93	63.17	9.55	59.35–66.99	2.49E-6^***^
	4	32.00	9.21	26.79–37.21	52.94	8.51	49.55–56.36	8.63E-7^***^
RUNX3		29.75	7.98	25.24–34.26	51.83	8.67	48.36–55.30	4.00E-8^***^
ETS1		39.17	15.07	30.64–47.69	75.79	11.67	71.16–80.42	4.05E-7^***^
RARG	1	36.30	10.17	30.54–42.06	50.20	11.30	45.68–54.72	5.21E-4^**^
	2	32.81	13.17	25.36–40.26	47.63	13.13	42.38–52.88	2.13E-3^*^
	3	35.96	12.34	28.98–42.94	50.34	12.86	45.19–55.49	1.77E-3^*^
	4	21.75	6.42	18.11–25.38	28.48	12.86	25.89–31.06	3.61E-3^*^
	5	20.78	7.52	16.52–25.03	28.36	7.59	25.32–31.40	4.70E-3^*^
	6	29.55	10.54	23.58–35.51	39.11	9.88	35.16–43.06	8.05E-3^*^
	7	24.58	8.83	19.59–29.58	36.80	7.42	33.83–39.77	2.90E-4^**^
CTNNB1		3.67	1.30	2.93–4.40	4.92	2.55	3.90–5.94	1.00E-2^*^
CDC25B	1	34.95	11.44	28.48–41.43	59.75	11.73	55.05–64.44	1.81E-6^***^
	2	42.94	12.88	35.65–50.22	72.23	12.79	67.11–77.34	8.73E-7^***^
	3	47.26	8.22	42.60–51.91	72.23	10.43	68.06–76.40	9.17E-9^***^
	4	45.03	7.85	40.58–49.47	70.52	10.64	66.27–74.78	3.07E-9^***^
	5	47.15	8.34	42.43–51.86	72.40	10.60	68.16–76.64	9.78E-9^***^
	6	43.70	9.12	38.54–48.86	66.58	12.52	61.57–71.58	4.11E-7^***^
	7	33.12	13.74	25.34–40.89	49.73	16.84	42.99–52.47	1.94E-3^*^
	8	17.38	14.99	8.90–25.86	23.58	12.42	18.61–28.55	0.12
PDCD1		35.25	11.84	28.55–41.95	65.13	11.46	60.54–69.71	1.82E-7^***^
CD247	1	72.50	16.42	63.21–81.79	96.83	6.11	94.39–99.28	1.44E-4^**^
	2	50.50	9.21	45.29–55.71	76.04	12.10	71.20–80.88	5.71E-8^***^
	3	53.83	9.85	48.26–59.41	81.00	10.03	76.99–85.01	4.29E-8^***^
	4	54.08	9.09	48.94–59.23	82.71	10.60	78.47–86.95	4.03E-9^***^
BTLA	1	61.33	11.79	54.66–68.00	84.75	8.29	81.43–88.07	5.77E-6^***^
	2	58.75	10.97	52.54–64.96	79.92	7.34	76.98–82.85	8.37E-6^***^
LTA	1	24.09	10.57	18.12–30.07	42.29	12.83	37.15–47.42	5.74E-5^***^
	2	18.13	9.41	12.80–23.45	34.04	10.85	29.69–38.38	6.09E-5^***^

The statistical significance: ^*^p-value < 0.01, ^**^p-value < 0.001, ^***^p-value < 0.0001.

### Methylation status validation and expression levels of network-based hypermethylated genes in KD patients

**Figure 3:**
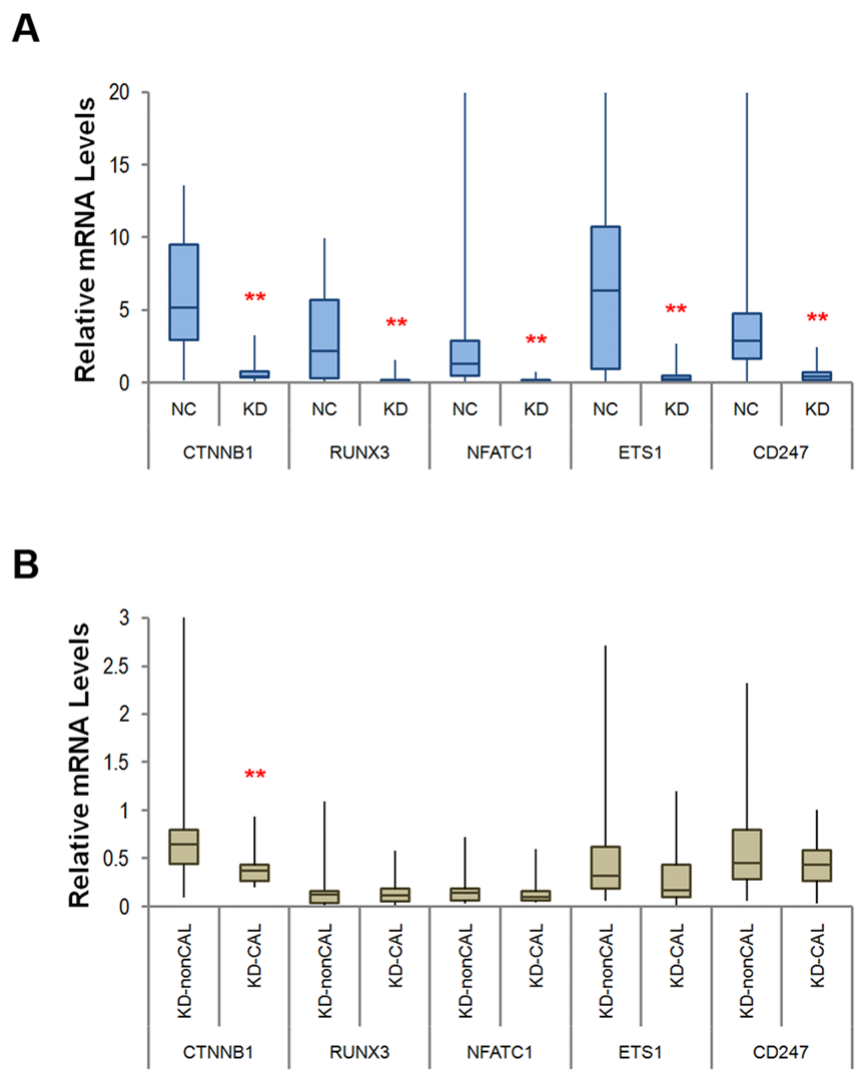
Box and whisker plots of the medium-level mRNA expression for the five network-based transcription regulators, measured using quantitative RT-PCR **(A)** The mRNA levels of all five genes were considerably lower in KD patients (n = 34) than in the normal controls (NC, n = 62); **(B)** Only the expression levels of β-catenin were significantly lower in KD patients with coronary artery lesions (CAL) when compared to non-CAL KD patients. ^**^p < 0.005 for each site according to the Mann–Whitney *U* test.

To validate the results from the network analysis, the methylation status of the aforementioned genes was detected using target-specific sequencing at CpG methylation sites pursuant to the array data via pyrosequencing. We validated 31 loci across the gene regions of the four major transcription regulators, as well as the cell cycle-related regulator cell division cycle 25B (CDC25B), the cardiac immune regulator programmed cell death 1 (PDCD1), and its three related effectors lymphotoxin α (LTA), B and T lymphocyte (BTLA), and CD247, of which 30 were found to be significantly hypermethylated among KD patients with ^*^p < 0.01 (Table 2, lower panel). We also studied the mRNA expression levels of the 10 network-associated genes with targeted re-sequencing by using next-generation sequencing technology, while the five transcription regulators NFATC1, ETS1, RUNX3, RARG, and CTNNB1 were also validated using quantitative RT-PCR in an independent validation set of subjects (n=34 for KD, n=62 for NC; see Table 1 lower panel). We adopted targeted RNA-Seq analysis to integrate next-generation sequencing and hybridization capture and performed oligonucleotide probes specific to the 11 selected genes using Illumina’s standard protocols and substituting the molecular indexing adaptors for standard ligation adaptors, as was previously described in the Materials and Methods section. The results of the targeted mRNA sequencing demonstrated that 10 of the validated networked genes were considerably lower in KD patients compared to the normal control subjects (p < 0.001, Supplementary Table 1). Furthermore, Figure 3A shows that the mRNA levels of the five selected transcription regulators validated by RT-PCR were significantly decreased in KD patients, but only the β-catenin mRNA expression remained significantly lower in KD patients with coronary artery lesions (CAL) when compared to non-CAL KD patients (Figure 3B). Overall, the epigenetic data and reduced expression levels of CTNNB1, the associated TFs (NFATC1, ETS1, RUNX3, and RARG), the correlated regulators CDC25B and PDCD1, and the related effectors LTA, BTLA, and CD247 may indicate that they have pivotal roles and a unique importance with regard to susceptibility to KD, as well as its cardiac pathogenesis.

### CTNNB1 knockdown significantly increases the *in vitro* expression of CD40 in human premonocytic THP-1 cells and CD40L in co-cultivated HCAEC

**Figure 4:**
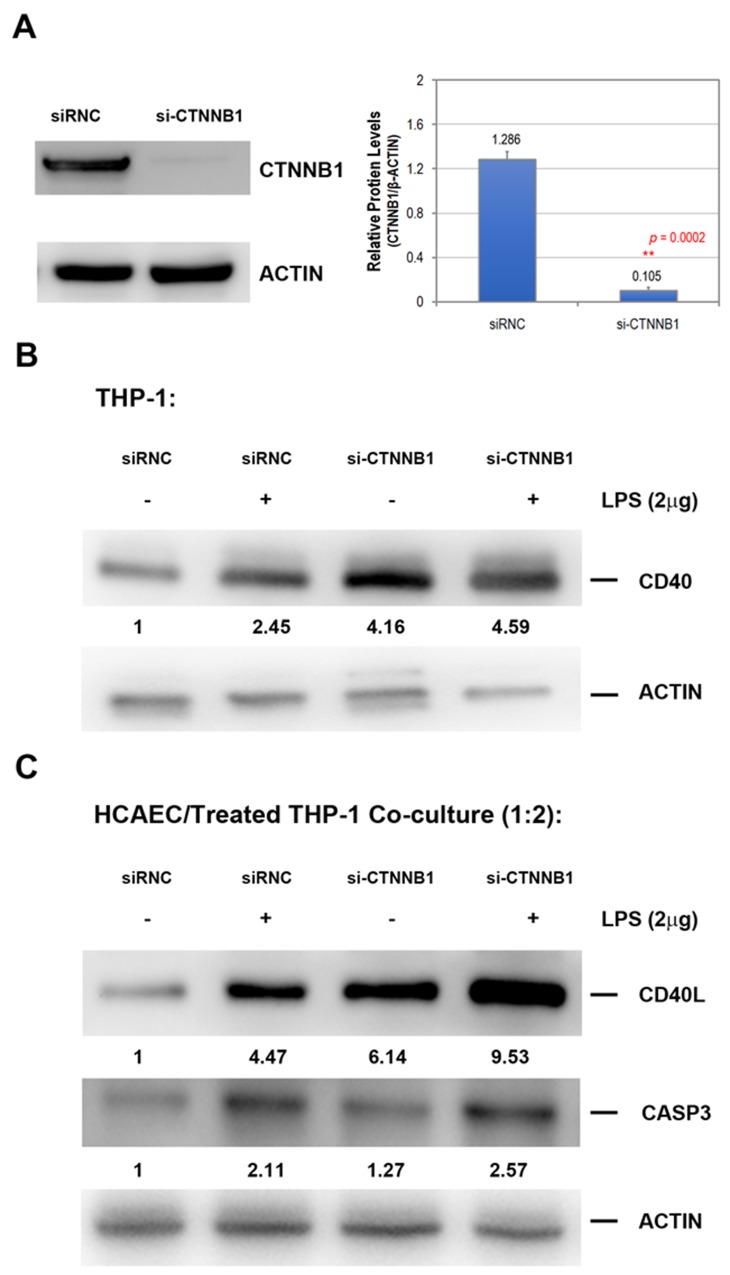
The knockdown of β-catenin on THP-1 considerably increases both CD40 and CD40L gene expression in cocultured endothelial cells **(A)** The human monocytic cell line THP-1 was transfected with control siRNA (siRNC, 3 ng/ml) or β-catenin siRNA (si-CTNNB1, 3 ng/ml) for 24 h and then harvested for Western blot analysis to evaluate the knockdown efficiency for the experiments. The expressed β-catenin protein determined by Western blot performed in triplicate. **(B)** The THP-1 monocytic cells were transfected with control siRNA (siRNC, 3 ng/ml) or β-catenin siRNA (si-CTNNB1, 3 ng/ml) for 24 h with control saline (-) or 2 μg/ml lipopolysaccharide (LPS) for another 24 h and then harvested for Western blot analysis. **(C)** THP-1 cells were initially transfected with control siRNA (siRNC, 3 ng/ml) or β-catenin siRNA (si-CTNNB1, 3 ng/ml) for 24 h and then exposed to saline (-) or LPS (+, 2 μg/ml) for another 24 h. Treated THP-1 cells were further added onto the cultured HCAEC with a ratio of 2:1 for another 8 h co-cultivating period. We measured the summary of the average density units under the blots in three independent experiments.

Researchers have previously indicated that the function of CD40 and CD40 ligand (CD40L) expression are correlated with KD susceptibility and the subsequent development of coronary artery lesions (CAL) [11]. To study the effects of β-catenin levels in monocytic cells, we knocked down β-catenin in a cultured THP-1 cell line using CTNNB1 siRNA for 24 h and treated it with 2 μg/mL lipopolysaccharide (LPS) or PBS for another 24 h, and then measured the protein levels of CD40 using Western blot analysis. THP-1 cells were grown to 60% confluence, followed by transfection with either human CTNNB1 siRNA (si-CTNNB1) or control siRNA (referred to as siRNC), as described in the Materials and Methods section. Protein lysates obtained from THP-1 cells after CTNNB1 knockdown showed a dramatic 10-fold suppression in total β-catenin protein compared to the normal control subjects (Figure 4A). Figure 4B demonstrates that the knockdown of both β-catenin and LPS increased the CD40 protein expression in THP-1 cells, and the combined treatment induced CD40 expression.

To investigate the effect of β-catenin-silenced THP-1 on endothelial cells, we cocultured primary coronary artery endothelial cells (HCAEC) with transfected and/or LPS-treated THP-1 cells for another 8 h, then washed out the suspended THP-1 cells, and analyzed the CD40 and CD40L expression in HCAEC. As shown in Figure 4C, β-catenin-silenced THP-1 largely increased both CD40 and CD40L expression in cocultured HCAEC. However, the up-regulation of CASP3 protein in HCAEC was only observed in cells cocultured with LPS-treated THP-1.

## DISCUSSION

Dynamic epigenetic alterations, such as the methylation status of CpG sites in gene regulatory regions, are recognized as a part of various human diseases. In this study, we identified a global DNA hypomethylation pattern and specific network interactions of hypermethylated genes in KD by using the HumanMethylation450 array at an increasing resolution. One of the most important signaling pathways, the wingless proteins (Wnt)/β-catenin pathway, influences cell destiny, proliferation, migration, and differentiation and is vital to embryonic development and tissue homeostasis via epigenetic changes [12]. This pivotal signaling pathway also modulates the regenerative process in body systems [13, 14]. Researchers have found that the Wnt/β-catenin pathway is activated in response to heart injuries to promote cardiac repair [15] and plays a vital role in cardiac hypertrophy and remodeling during the heart’s development [16, 17]. In this study, we identified a cluster of β-catenin with four transcriptional regulators: RARG, ETS1, NFATC1, and RUNX3 from hypermethylated DMRs in KD patients. These genes were considerably decreased in the blood cells of KD patients. Nevertheless, the interpretation of β-catenin’s function in KD pathogenesis may provide a clue about how the relatively few DMRs with hypermethylated CpG loci occurring among this cluster positively correlate with CAL symptoms among KD patients.

New evidence has shown that endothelial dysfunction caused by the vigorous development of immune responses is vital to the development of CAL in KD patients [18–20]. The interaction between CD40L and CD40 is critical for activating the immune system and enhancing pro-inflammatory cytokines and vascular endothelial cells’ interaction with immune cells [21–23]. Our previous studies have also shown that an increased expression of CD40L on CD4^+^ T-cells correlated with susceptibility, CAL formation, and disease severity of KD [11]. In the current study, we found that knockdown of β-catenin on THP-1 significantly increases CD40 and CD40L expression in cocultured HCAEC. Off-target effects may play a role in the synthetic siRNA used for β-catenin silencing which lead to increased CD40 expression in THP-1 cells. Therefore, we verified the CTNNB1 gene silencing results on CD40 expression by using another siRNA product, which targets different positions in CTNNB1 transcript (sc-29209, purchased from Santa Cruz Inc.), and observed similar knockdown efficiency in reducing β-catenin protein expression that also significantly enhanced CD40 levels in THP-1 cells as well as CD40/CD40L expression in cocultivated HCAEC. Furthermore, the additive effect of β-catenin silencing and LPS on THP-1 for the HCAEC-expressed CD40L protein levels may be caused by activating parallel signaling pathways. LPS is an outer membrane molecule of Gram-negative bacteria and induces innate immune responses and pro-inflammatory polarization (M1) in macrophages through TLR4 signaling [24, 25]. M1 macrophages secrete inflammatory cytokines and are known to be the major source of reactive oxygen species (ROS) in atherosclerotic lesions that may accelerate disease progression by promoting atherogenic responses in endothelial and vascular smooth muscle cells [25, 26]. Although M2 polarized macrophages represent an anti-inflammatory phenotype and appear critical for balancing inflammation, a recent study found that intracellular nicotinamide phosphoribosyltransferase (iNAMPT) exerted anti-atherogenic effects on low-density lipoprotein receptor-deficient mouse model while its extracellular pro-inflammatory role has been widely studied in association with several metabolic and inflammatory disorders [27]. A previous Apo E-deficient mice study also demonstrated that the presence of both M1- and M2-polarized macrophages in the arterial wall may contribute to distinct stages of inflammation in atherosclerotic plaque formation [28]. The results undoubtedly reflect the complexity of macrophage responses in aortic disease progression. CD40L (CD154) belongs to the tumor necrosis factor (TNF) superfamily, and the interaction with its receptor CD40 in a wide range of cell types in the vessel wall, thus leading to various inflammatory processes, has emerged as an important contributor to the inflammatory and atherogenic effects *in vitro* and *in vivo* [29–34]. CD40L has been shown to interact with CD40 on monocytes, macrophages, and endothelial cells, which induces intracellular signaling events, including the activation of mitogen-activated protein kinases (MAPKs), nuclear factor-κB (NF-κB), and the subsequent expression of genes encoding IL-6, TNFα, IL-8, inducible nitric oxide synthase (iNOS), and IL-12p40 [35–37]. CD40L has been shown to increase the expression of its receptor CD40 in endothelial cells, and some studies have indicated that the CD40L-induced CD40 expression contributes to endothelial dysfunction and vascular diseases [38, 39]. Therefore, the increased expression of CD40 through β-catenin silencing in THP-1 could be the result of a secondary response via the paracrine effect of CD40L. Of particular note, the expression of CASP3 in HCAEC, which is another susceptible gene for KD, was induced only by culturing LPS-treated THP-1, thus indicating a new mechanism through which endogenous suppression of β-catenin can additively enhance locoregional pro-inflammatory effects on innate immune cells and influence the pathogenic interaction with surrounding ECs in KD via epigenetic modulation. However, the role of β-catenin and its underlying mechanism with regard to CAL in KD still require additional research.

In this study, we also discovered the two associated regulators, PDCD1 and CDC25B, and the three associated effectors, CD247, BTLA, and LTA, in SNEA, all of which interacted with the putative β-catenin-associated cluster and were also significantly hypermethylated in several promoter CpG loci; furthermore, their mRNA expressions were decreased in KD. For the predicted regulator PDCD1, a co-inhibitory member of the CD28/CTLA-4 molecules expressed on lymphocytes, its role is to maintain the T cell unresponsiveness that is demonstrated in various experimental models of autoimmune and allergic inflammation [40–43]. The blockade of the programmed death receptor has been commonly researched and been found to exhibit enhanced rejection in transplants while promoting inflammation in various diseases, including stroke, vasculitis, encephalomyelitis, viral myocarditis, and atherosclerosis [44–51]. Of particular interest, PDCD1 has been observed as one of the genetic predispositions of KD-like features in PDCD1 knockout mice [52]. In the current study, we found that a specific regulatory region of PDCD1 was significantly hypermethylated in KD. This DMR, which ranges among the TSS1500 from the start codon of the PDCD1 gene, has also been observed in HIV-specific CD8 T cells, in which PDCD1 expression in prolonged virus exposure is increased [53]. This study is the first to reveal a correlation between PDCD1 down-regulation via epigenetic control and susceptibility to KD.

Furthermore, the identified effector CD247, also known as TCR/CD3-zeta, which encodes a transmembrane protein in the TCR complex, has been found to be responsible for the expression of inhibitory CTLA-4 molecules [54]. Defects in CD247 signaling result in deficient CTLA-4 expression and impaired CD4^+^CD25^+^Foxp3 regulatory T cells. In this study, we discovered four significantly hypermethylated CpG loci on the CD247 promoter in KD, which demonstrates a correlation between CD247 down-regulation and susceptibility to KD, that may be the result of DNA methylation. Moreover, we identified three hypermethylated CpG loci on the prompter region of the immune inhibitory receptor BTLA gene related to KD susceptibility. Taken together, our data indicates that the epigenetic down-regulation of BTLA, PDCD1, and CD247 in KD patients may enforce a poised impairment of immune suppression and exert a strong inflammatory storm in the organs. WNT signaling has been shown to be essential for T cell development in thymus [55–58], and the knockdown of β-catenin decreased T_H_2 linage commitment and cytokine production in association with decreased GATA3 expression [59]. In our study, we also observed reduced GATA3 mRNA expression, as well as an increased proportion of CD4^-^CD8^-^CD44^+^ T lymphoid cells in the peripheral blood of KD patients (data not shown). The increase of double-negative T lymphoid cells implies that the epigenetic down-regulation of β-catenin may affect the development of early T cell commitment in KD patients. In KD, vascular inflammation leads to the permanent damage of arteries, and the development of aneurysms is usually associated with macrophage accumulation, which also points towards a specific immune cell-endothelial cell interaction. A recent study demonstrated that serum from KD patients induced endothelial-to-mesenchymal transition (EndoMT) via suppression of the KLF4/miR-483 axis in primary human umbilical vein endothelial cells [60]. However, how the differential impacts of immune cells transmit acute inflammatory signals to vascular endothelial cells and cause persistent vessel wall abnormalities in KD require further research.

Therefore, the CpG locus and the expression levels of β-catenin may be pivotal in determining the disease severity of KD patients. Additional research into the underlying mechanism of the β-catenin-mediated signaling involved in the systemic cause and development of coronary artery injuries in KD is necessary and can potentially improve patient outcomes.

## MATERIALS AND METHODS

### Participants

The Institutional Review Board of the Chang Gung Memorial Hospital (No. 101-0680A3) approved this study, and we obtained informed consent from all of the participating children’s parents or guardians. All participants were first given IVIG (2 g/kg) over a 12-h period. We excluded any patients whose symptoms did not completely fit the diagnostic criteria of KD set forth by the American Heart Association, as in our previous studies [18, 61]. This study also included blood samples from age-matched non-KD control subjects (with no history of KD) for comparison.

### DNA extraction and bisulfite conversion of genomic DNA (gDNA)

In order to extract DNA, we first treated the collected blood cells with a 0.5% SDS lysis buffer and then with protease K (1 mg/ml) for 4 h at 60°C to digest the nuclear protein. Complete genomic DNA was harvested using the Gentra extraction kit, followed by precipitation with 70% alcohol. NanoDrop ND-1000 (NanoDrop Technologies, Wilmington, DE, USA) was used to quantify 500 ng of gDNA from each sample, followed by bisulfite conversion of the gDNA samples using the Zymo EZ DNA Methylation kit (Zymo Research Corporation, Orange, CA, USA). The bisulfite-converted gDNA was eluted and stored at -20°C until ready for use.

### Genomic CpG methylation profiling

Infinium HumanMethylation450 BeadChip (Illumina, San Diego, CA, USA) was used to carry out genome-wide DNA methylation patterns across 485,577 CpG loci. This panel was aimed at 96% of known CpG loci and included nearly all NCBI consensus coding sequence (CDS) genes. On average, 17 CpG sites per gene were distributed across the 5′ flanking region of the transcription start sites TSS1500 and TSS200 (promoter), 5′ untranslated regions (UTR), first exon, gene body, and the 3’UTR. Additional specifications related to the array platform can be found online at http://support.illumina.com/array/array_kits/infinium_humanmethylation450_beadchip_kit.html.

Bisulfate-converted DNA (BCD, 500 ng) was used per BeadChip for hybridization in accordance with the protocol and instructions of Infinium HD methylation. After completing the whole-genome amplification step and then controlled enzymatic end-point fragmentation, we precipitated and re-suspended the BCD samples for 16 h at 48°C for hybridization. During this process, the DNA molecules were annealed to two bead types with methylated loci (C)-specific and unmethylated loci (T)-specific oligomers. Afterward, the non-hybridized or mis-hybridized DNA was washed away, followed by single-base extension using 2,4-dinitrophrnol- and biotin-labeled ddNTPs and the annealed BCD as templates. We then fluorescently stained and scanned the array and extracted the intensities of the beads using GenomeStudio methylation module (v.1.9.0) software.

The methylation score of the interrogated CpG locus was represented with a beta value that was determined according to the ratio of normalized probe intensity between methylated and unmethylated signals (C/C+T). Beta values varied between 0 (unmethylated) and 1 (fully methylated). The average delta-beta values demonstrated the differential methylation between samples from KD patients and those from normal control subjects. The Pearson correlation coefficient was used as a distance metric to create the hierarchical clustering heat map of the differentially methylated probes in the Partek Genomic Suite (Partek Inc., USA).

### Pyrosequencing analysis

We applied pyrosequencing to validate the methylation status by using a PyroMark Q24 instrument (Qiagen, Germany) and performed the subsequent quantification of methylation levels using its software (ver. 1.0.10). We prepared the BCD from 0.5 μg genomic DNA using an EZ DNA methylation kit (Zymo Research) and then re-suspended it in 20 μl of Tris buffer (10mM), which was then amplified by polymerase chain reaction (PCR) in the regions of interest with the PyroMark PCR kit (Qiagen, Germany) in accordance with the recommended protocol. Source sequences for the predesigned and customized pyrosequencing assays are provided in Supplementary Table 2. The sequencing results used to calculate the percentage of methylated cytosines at each given locus were analyzed using the previously described software [62].

### RNA isolation, targeted RNA-Seq, and quantitative RT-PCR

Complete RNA from the white blood cells (WBC) of the participants was isolated using a FavorPrep Blood/Cultured Cell Total RNA Mini kit (#FABRK001-1, Favorgen Biotech Corporation, Ping-Tung, Taiwan). We targeted RNA-Seq using customized TruSeq Targeted RNA Expression Kits and MiSeq System from Illumina (San Diego, CA, USA). Both the sample preparation and the RNA-Seq procedure were performed pursuant to the manufacturer’s instructions. The customized panel genes used in the TruSeq Targeted RNA Expression are listed in Supplementary Table 3. The quantitative RT-PCR reaction was carried out with an ABI 7500 Fast Real-Time PCR System using the ABI TaqMan Fast Universal PCR master mix. Normalization was determined with human β-actin probe (ACTB, P/N: 4331128, ID: Hs01060665_g1). In order to detect expression level, we obtained the TaqMan probes for β-catenin (CTNNB1, P/N: 4331128, ID: Hs00355049_m1), NFATC1 (P/N: 4331128, ID: Hs00542678_m1), RUNX3 (P/N: 4331128, ID: Hs00231709_m1), ETS1 (P/N: 4331128, ID: Hs00428293_m1), and RARG (P/N: 4331128, ID: Hs01559234_m1) from Thermo Fisher Scientific. We verified the comparative RT-PCR data three times and calculated the fold increase using the comparative 2^-ΔΔCt^ method.

### Cell cultivation and *in vitro* vasculitis model

We grew human coronary artery endothelial cells (HCAEC) in an EGM-2 MV BulletKit (#CC-3202, including basal medium [CC-3156], supplements, and growth factors [CC-4147], Lonza Group AG, Basel, Switzerland) at 37°C in a humid environment with 5% CO_2_. Human monocytic leukemia cells THP-1 were cultured in RPMI containing 10% FCS, 100 U/ml penicillin and 100 μg/ml streptomycin at 37°C in 5% CO_2._ For the experimental assays, we transfected cells using the GenMute siRNA Transfection Reagent (#SL100568, SignaGen Laboratories, Rockville, MD, USA) with siRNA negative control (siRNC, 25nM) and CTNNB1 siRNA (10nM) from Sigma-Aldrich (St. Louis, MO, U.S.) according to the manufacturer’s instructions.

For contact cocultivation of monocytes and endothelial cells, 2 mL aliquots of THP-1 cells (1 × 10^6^ cells/well) were added to 6-well plates onto confluent HCAEC layers (5 × 10^5^ cells/well) in ECM. The ratio of the THP-1 cells and the HCAEC monolayer is 2:1 monocytes to ECs. We performed experiments with contact cocultures in the presence or absence of 2 μg/ml Lipopolysaccharide (LPS) for 24 h. To determine the role of suppressed CTNNB1 in monocyte-endothelial cell interactions, THP-1 cells (1 × 10^6^ cells/mL) were transfected with CTNNB1 siRNA or control siRNA for 24 h; HCAEC (5 × 10^5^ cells/mL) were directly co-cultivated with THP-1 cells for 8 h.

### Western blot analysis

All proteins were homogenized with a loading buffer, separated by 10% SDS-PAGE, and transferred to nitrocellulose membranes, which were then probed overnight with primary antibodies at 4°C and incubated for 1 hour with respective conjugated secondary antibodies (1:2000, Cell Signaling Technology, Billerica, MA, USA). Immunoreactive proteins were visualized using ECL Western blotting detection reagents (Millipore) and quantitated using a G:BOX iChemi XL imaging system (J&H Technology Co. Ltd.). The Western blotting reactivity of CTNNB1 (β-catenin, sc-7199), CD40L (CD154, sc-978), CD40 (sc-975), and CASP3 (sc-7148) were measured using primary anti-human antibodies (Santa Cruz Biotechnology, Dallas, TX, USA) from lysates of siRNC-, CTNNB1 siRNA- transfected, and/or LPS-treated endothelial cells, respectively.

### Statistical analysis

Continuous data are presented as median and range and compared among groups using the Mann–Whitney *U* test. We performed statistical analysis using the SPSS software package for Windows (SPSS 15.0 for Windows; SPSS Inc., Chicago, IL, USA). To discover the molecular facts and biological context of the epigenetic changes, we analyzed data using Ariadne’s Pathway Studio (Elsevier B.V., Amsterdam, Netherlands) to establish sub-network enrichment analysis with direct interactions. All p-values were derived from 2-tailed tests, and a value of <0.01 was considered statistically significantly.

### Availability of array data

All normalized and raw 450K methylation array data were submitted to and are available in the Gene Expression Omnibus repository (GEO, Bethesda, MD, USA; accession GSE84624: http://www.ncbi.nlm.nih.gov/geo/query/acc.cgi?acc=GSE84624).

## SUPPLEMENTARY MATERIALS TABLES






