# Supplementary material for: The human blood DNA methylome identifies crucial role of β-catenin in the pathogenesis of Kawasaki disease
Source: Oncotarget. 2018 Jun 19;9(47):28337–50. doi: 10.18632/oncotarget.25305 (PMC6033340; doi:10.18632/oncotarget.25305)
Supplement: Supplementary file 1 [file oncotarget-09-28337-s001.pdf]

The human blood DNA methylome identifies crucial role of β-catenin in the pathogenesis of Kawasaki disease

SUPPLEMENTARY MATERIALS

Supplementary Table 1: Expression validation of network-based genes through targeted RNA-Seq analysis

Ensembl ID	Gene symbol	Description	Mean reads	Ratio	Adjusted p-value
ENSG00000168036	CTNNB1	catenin (cadherin-associated protein), beta 1, 88kDa	100.67	0.3724	8.37E-08
ENSG00000131196	NFATC1	nuclear factor of activated T-cells, cytoplasmic, calcineurin-dependent 1	76.5	0.4297	7.53E-07
ENSG00000134954	ETS1	v-ets erythroblastosis virus E26 oncogene homolog 1	141.1	0.2708	4.34E-12
ENSG00000172819	RARG	retinoic acid receptor, gamma	184.7	0.3274	1.62E-04
ENSG00000101224	CDC25B	cell division cycle 25B	49400.6	0.4732	2.03E-18
ENSG00000020633	RUNX3	Runt-related transcription factor 3	130.5	0.2617	3.02E-07
ENSG00000198821	CD247	CD247 molecule	1273.9	0.6621	4.28E-13
ENSG00000226979	LTA	lymphotoxin alpha (TNF superfamily, member 1)	426	0.6309	3.57E-11
ENSG00000186265	BTLA	B and T lymphocyte associated	1065.8	0.3804	0.000725
ENSG00000188389	PDCD1	programmed cell death 1	1483.1	0.4764	1.44E-06
ENSG00000165025	SYK	spleen associated tyrosine kinase	N.A.		

N.A.: not available.

Supplementary Table 2: Hypermethylated CpG loci with source sequences selected using network-based enrichment analysis.

See Supplementary File 1

Supplementary Table 3: Probe data of 11 targeted genes with hypermethylated CpG loci selected using network-based enrichment analysis predesigned for RNA-Seq.

See Supplementary File 2
