# Supplementary material for: The human blood DNA methylome identifies crucial role of β-catenin in the pathogenesis of Kawasaki disease
Source: Oncotarget. 2018 Jun 19;9(47):28337–50. doi: 10.18632/oncotarget.25305 (PMC6033340; doi:10.18632/oncotarget.25305)
Supplement: Supplementary file 2 [file oncotarget-09-28337-s002.pdf]

Supplementary Table 2. Hypermethylated CpG loci with source sequences selected using network-based enrichment analysis.

CpG loci ID	Gene Symbol	Chr	Source Sequence	Coordinate in Build 36	Genomic Location	Strand	Functional Location	Δ-Beta	Adjusted p-value
cg24157392	BTLA	3	CGGTAAGCAAATGTAACAGCTTGGTTTCATCTACGATTACCCACTTCGTA	113700663	3:113700663	F	Body	0.25195	1.60E-08
cg09032544	CD247	1	CGGAAAATGTCAGCCAGGCCATGTTTCGTGCTGCGACGTTCTAGTCCCTT	165753919	1:165753919	R	Body	0.31832	7.70E-04
cg07786657		1	TCCCCTACACATACACAAAGAGTTTCTAGAAGTTCCCTGCCGTCGACACG	165754257	1:165754257	R	Body	0.36159	5.60E-05
cg14165142	CDC25B	20	AGAGGGTGGGGCCCTCTACGGAGATGAGATCTTACCTATGTAAAACCACG	3726655	20:3726655	R	Body	0.25253	1.10E-08
cg02737268		20	CCGTGTTACAGGAGACCACCTGCCACGCGGAGCTTTTGTGCATCTACGCG	3728182	20:3728182	F	Body	0.42308	1.90E-09
cg15421087	CTNNB1	3	CGGCGCGGGGACTACTTTCCACCGCCCCCTCGCGCCCCGCCCCTTGTCCT	41215859	3:41215859	F	TSS200	0.25553	3.20E-05
cg03295554	ETS1	11	CGGCTGATCCAGAACCAGTTTATGACTCACAGATGTTGATTTACTGGTAT	127900660	11:127900660	F	Body	0.26371	1.00E-05
cg26404422		11	CGAAGTCACCAGCCAGTAAAAACCACTGCAAAGAAGGGAGAAGAAGCTGC	127872220	11:127872220	F	Body	0.26570	3.40E-04
cg26348243	LTA	6	CGCGGCGGAAGACAGACCTCCCGCCCTGGGAGAGAGCACCCCCCGACCCC	31648440	6:31648440	R	5'UTR	0.28315	7.60E-04
cg14441276		6	CTCAGCCAAGGGTGCAGAGATGTTATATATGATTGCTCTTCAGGGAACCG	31647714	6:31647714	R	TSS1500;TSS200	0.25889	2.40E-06
cg02402436		6	GATGGGGCAGGAGAGCCTCACCTGCTGTGCGGAGCCCCTGGGCCCGGACG	31648030	6:31648030	F	TSS200;1stExon;5'UTR	0.26393	1.70E-09
cg21999229		6	CGCTGCCACTGCCGCTTCCTCTATAAAGGGACCTGAGCGTCCGGGCCCAG	31647993	6:31647993	F	TSS200;1stExon;5'UTR	0.26700	1.50E-03
cg16219283		6	GCTCAGGTCCCTTTATAGAGGAAGCGGCAGTGGCAGCGTGGCAGGCAGCG	31647981	6:31647981	F	TSS200;1stExon;5'UTR	0.27092	9.00E-09
cg14597739		6	CGCCCGCTGCCTGCCACGCTGCCACTGCCGCTTCCTCTATAAAGGGACCT	31647977	6:31647977	F	TSS200;1stExo	0.28225	2.50E-05

							n;5'UTR		
cg17169196		6	TGTGCGGAGCCCCTGGGCCCGGACGCTCAGGTCCCTTTATAGAGGAAGCG	31648005	6:31648005	F	TSS200;1stExon;5'UTR	0.28398	6.90E-12
cg09621572		6	CGGGCCCCAGCCCCGACCTAGAACCCGCCCGCTGCCTGCCACGCTGCCAC	31647952	6:31647952	F	TSS200;1stExon;5'UTR	0.33790	3.70E-08
cg14437551		6	AGAGGAAGCGGCAGTGGCAGCGTGGCAGGCAGCGGGCGGGTTCTAGGTCG	31647965	6:31647965	F	TSS200;1stExon;5'UTR	0.36865	5.10E-06
cg22324981	NFATC1	18	CGCCTACGGCTTGTTTACGCACAGCTAGTGAGAGCTTGCGTGGCCCCAGC	75384481	18:75384481	F	Body	0.30353	4.00E-10
cg16308790		18	GCTTCAGCTCCCCTGGGGCAGCTCTGATGCGGGGCAAAGGCAGGAAGACG	75326961	18:75326961	R	Body	0.28664	4.50E-06
cg03889044	PDCD1	2	ATGCTCTTTTTCCACTGTTTCGGTGCTTTAATGTTTTCCCTTCAGAGCCG	242450772	2:242450772	F	TSS1500	0.26858	3.10E-10
cg17322655		2	TGGCCGCGCCTCGCAGACATCATCTTTGATGCTCTTTTTCCACTGTTTCG	242450800	2:242450800	F	TSS1500	0.27499	1.90E-08
cg20805133		2	CGAGAGCTTCCTCGCCGTGGCCGCGCCTCGCAGACATCATCTTTGATGCT	242450865	2:242450865	R	TSS1500	0.36842	6.60E-05
cg09993145	RUNX3	1	CGCCCACTGCAACCGCTTTCAGTTCTGTTTCTTGGGCCGCGTGCTGAGGC	25164492	1:25164492	R	TSS1500	0.38990	4.20E-04
cg13461622		1	CGCTTCTGTTGAGAATTTGTGGCTAGACATTCCTGTGGGACCGGGAATCC	25163972	1:25163972	R	1stExon;5'UTR	0.27998	2.20E-09
cg03961551		1	TTTCCCAGTCAGCAGGATGGGCACTGCAGATGTGTGTCTGCATGCCAGCG	25124317	1:25124317	R	Body	0.29300	4.30E-06
cg14054883	SYK	9	AAAACCTTTTCCTTGTTCATTGAGGAATGAGACTGTGTTAGGCTGTCACG	92659288	9:92659288	R	Body	0.26680	1.60E-08
cg20059012	RARG	12	CGTGTGCCCCTGCGTGGCTGCGGGGGCGGTTTTGGAAAGTGCTGTGTTCG	51899421	12:51899421	F	Body	0.27935	7.70E-07
