# Supplementary material for: The human blood DNA methylome identifies crucial role of β-catenin in the pathogenesis of Kawasaki disease
Source: Oncotarget. 2018 Jun 19;9(47):28337–50. doi: 10.18632/oncotarget.25305 (PMC6033340; doi:10.18632/oncotarget.25305)
Supplement: Supplementary file 3 [file oncotarget-09-28337-s003.pdf]

Supplementary Table 3. Probe data of 11 targeted genes with hypermethylated CpG loci selected using network-based enrichment analysis predesigned for RNA-Seq.

Gene Name	Transcript ID	Build ID	Chr	Start Position	End Position	ULSO Sequence	ULSO Hits	DLSO Sequence	DLSO Hits	Probe Strand
RUNX3	NM_001031680	hg19	chr1	25256102	25254180	CAAGACGCTGCCCGTCGCCTTCAA	1	GACGTGCCGGATGGTACGGTGGTGACT	1	MINUS
ETS1	NM_001143820	hg19	chr11	128333436	128332437	GGCTGGGAATTCAAACTTTCTGACC	1	AGATGGGGAAAGAGGAAAAACAAACC	3	MINUS
ETS1	NM_001143820	hg19	chr11	128426220	128360447	GGAAGTTCCTACTGGTCTTGAACACTG	1	TATGGAATGTGCAGATGTCCCACT	3	MINUS
CDC25B	NM_004358	hg19	chr20	3777344	3778339	ACCCAGACCATGCACGACCTCG	1	CAGCCGCCTGACGCACCTATCCCTGT	1	PLUS
PDCD1	NM_005018	hg19	chr2	242800987	242800924	CAGATCCCACAGGCGCCCTGGCCAGT	1	GTGCTACAACTGGGCTGGCGGCCAGGAT	1	MINUS
CDC25B	NM_021872	hg19	chr20	3781120	3781655	CCATCAGACGCTTCCAGTCTATGC	2	TTCAAGATGCCATGGAAGCCCACACAT	1	PLUS
CDC25B	NM_021873	hg19	chr20	3785295	3785492	GGCGGCTACAAGGAGTTCTTCCCTCA	1	ACTTCTGTGAACCCCAGGACTACC	1	PLUS
NFATC1	NM_172387	hg19	chr18	77246905	77287566	CTGAAGAGTTGGACCAGTTGTACCT	1	AATGACCTCTCCAGCACGAGCA	8	PLUS
NFATC1	NM_172388	hg19	chr18	77156327	77193619	GGCACCATGAAGTCAGCGGAGGAA	2	CCTGGACTGGCAGCTGCCGTCCCACT	1	PLUS
NFATC1	NM_172389	hg19	chr18	77193711	77208804	GTCGGCCGGAGGACACCCCATCGT	1	CTGCATGGCTACTTGGAGAATGA	4	PLUS
CTNNB1	NM_001098210	hg19	chr3	41241134	41265540	CGCGGCGGGAGGAGCCTGTTCCCCTGA	1	GGTATTTGAAGTATACCATACAACTGTTT	1	PLUS
CD247	NM_198053	hg19	chr1	167487681	167409965	ACCGCGGCCATCCTGCAGGCACAGTT	2	GCCTGCTGGATCCCAAACTCTGCTA	2	MINUS
CD247	NM_198053	hg19	chr1	167407845	167404646	AAGAGACGTGGCCGGGACCCTGAGAT	1	AGAGAAGGAAGAACCCTCAGGAAGG	5	MINUS
RARG	NM_000966	hg19	chr12	53606893	53605606	GACCTCCGGGGCATCAGCACTAA	1	TTACTCTGAAGATGGAGATTCCAGGCC	1	MINUS
RARG	NM_001243731	hg19	chr12	53624980	53609190	GAAGCCAGTCTCTGCAGGCGGCCA	3	TTTCGCCGAAGCATCCAGAAGAA	5	MINUS
RARG	NM_000966	hg19	chr12	53625786	53624976	CGCCGCCTCCCTTCCCCCTCCCACTC	9	AACCTGACCCAGTATGTAGAAGC	1	MINUS
SYK	NM_003177	hg19	chr9	93650877	93657848	GTACGATCTCATGAATCTGTGCTGGA	1	GATTCGCAGCAGTGGAACTGCG	6	PLUS

SYK	NM_001174167	hg19	chr9	93589831	93606182	CCAGATCTGCGTTTGAATCCAGGAAA	1	TGCCCTCCGGCCCCTGAAGCAT	9	PLUS
SYK	NM_001174168	hg19	chr9	93627351	93636514	GTCCACAACTTCCAGGTTCCCAT	5	CCTCCCCTGCCCAAGGGAACCGGCAAGA	1	PLUS
GAPDH	NM_002046	hg19	chr12	6647132	6647310	TCAACGACCACTTTGTCAAGCTCA	37	CAGCAACAGGGTGGTGGACCTCA	46	PLUS
LTA	NM_001159740_2	hg19	chr6	31540041	31540542	GGGACCTGAGCGTCCGGGCCCA	6	CCATGACACCACCTGAACGTCTCTT	1	PLUS
LTA	NM_000595_3	hg19	chr6	31541751	31541808	TGGGGCCTAGATCCACACACAGAGGAA	1	GCACATGGAGGAGCTTGGGGGATGA	1	PLUS
BTLA	NM_181780	hg19	chr3	112190093	112188618	TTTCTGCCTGTTCTGCTGCCTGAGAA	2	AAATGAACTCTCTGACACAGCAGGAAG	1	MINUS
BTLA	NM_181780	hg19	chr3	112218150	112198578	TAATCCCATATCTGGACATCTGGAACA	1	CATGTGATGTACAGCTTTATATAAAGAGAC	1	MINUS

ULSO: upstream locus-specific oligonucleotides; DLSO: downstream locus-specific oligonucleotides.
